# P-949. The Success of Rapid-Fire Journal Club in Achieving Doctor of Pharmacy Educational Outcomes

**DOI:** 10.1093/ofid/ofae631.1139

**Published:** 2025-01-29

**Authors:** Nicole Bradley, Yuman Lee

**Affiliations:** St. John's University, Queens, New York; St. John's University, Queens, New York

## Abstract

**Background:**

Rapid-fire journal club is an activity in the infectious diseases elective for pharmacy students in their third professional year. In small groups, students were tasked with completing a journal club on an assigned study evaluating new antibiotics for treatment of resistant gram-negative infections. Students were given 1 week to prepare their presentation, and 10 minutes deliver their presentation. The goal of rapid-fire journal club was to engage pharmacy students in primary literature relating to new antibiotic agents and differentiate how each of the agents is best used in practice. The purpose of this study was to assess the impact of rapid-fire journal club on achieving Curriculum Outcomes and Entrustable Professional Activities (COEPA) within a Doctor of Pharmacy Program.

**Methods:**

Student feedback was anonymously collected via an electronic survey. Students evaluated the impact of rapid-fire journal club across the 3 domains of the American Association of Colleges of Pharmacy (AACP) COEPA educational outcomes (Knowledge, Skills, and Attitudes) on a Likert scale from strongly disagree (1) to strongly agree (5). Student perceptions and feedback on the activity were also collected.

**Results:**

29 of the 38 students who participated responded. Majority strongly agreed or agreed that rapid-fire journal club allowed them to build foundational knowledge of antibiotics for resistant gram-negative infections (27/29, 93.1%), solve problems (21/29, 72.4%), improve communication among colleagues (24/29, 82.7%), understand the role of primary literature in improving antibiotic use (26/29, 89.7%), support shared goals on a team (25/29, 86.2%), and build and maintain trust among colleagues (25/29, 86.2%).

Majority of students rated the activity a 3 out of 5 in difficulty, found the activity engaging, and enjoyed participating.

**Conclusion:**

Rapid-fire journal allowed pharmacy students to achieve key Doctor of Pharmacy educational outcomes across the 3 COEPA domains. The activity was positively received by students and provided them with a unique activity within the classroom.

**Disclosures:**

**All Authors**: No reported disclosures

